# Exome Sequencing Identifies the Extremely Rare *ITGAV* and *FN1* Variants in Early Onset Inflammatory Bowel Disease Patients

**DOI:** 10.3389/fped.2022.895074

**Published:** 2022-05-26

**Authors:** Huda Husain Al-Numan, Rana Mohammed Jan, Najla bint Saud Al-Saud, Omran M. Rashidi, Nuha Mohammad Alrayes, Hadeel A. Alsufyani, Abdulrahman Mujalli, Noor Ahmad Shaik, Mahmoud Hisham Mosli, Ramu Elango, Omar I. Saadah, Babajan Banaganapalli

**Affiliations:** ^1^Department of Biological Sciences, Faculty of Science, King Abdulaziz University, Jeddah, Saudi Arabia; ^2^Princess Al-Jawhara Al-Brahim Center of Excellence in Research of Hereditary Disorders, King Abdulaziz University, Jeddah, Saudi Arabia; ^3^Saudi Ajal for Health Services, Riyadh, Saudi Arabia; ^4^Department of Medical Laboratory Sciences, Faculty of Applied Medical Sciences, King Abdulaziz University, Jeddah, Saudi Arabia; ^5^Department of Medical Physiology, Faculty of Medicine, King Abdulaziz University Hospital, Jeddah, Saudi Arabia; ^6^Department of Laboratory Medicine, Faculty of Applied Medical Sciences, Umm Al-Qura University, Makkah, Saudi Arabia; ^7^Department of Genetic Medicine, Faculty of Medicine, King Abdulaziz University, Jeddah, Saudi Arabia; ^8^Department of Internal Medicine, King Abdulaziz University, Jeddah, Saudi Arabia; ^9^Inflammatory Bowel Disease Research Group, King Abdulaziz University, Jeddah, Saudi Arabia; ^10^Department of Pediatrics, Faculty of Medicine, King Abdulaziz University, Jeddah, Saudi Arabia

**Keywords:** early onset-inflammatory bowel disease, WES, monogenic diseases, consanguineous, digenic inheritance, gene expression

## Abstract

**Background:**

Molecular diagnosis of early onset inflammatory bowel disease (IBD) is very important for adopting suitable treatment strategies. Owing to the sparse data available, this study aims to identify the molecular basis of early onset IBD in Arab patients.

**Methods:**

A consanguineous Arab family with monozygotic twins presenting early onset IBD was screened by whole exome sequencing (WES). The variants functional characterization was performed by a series of computational biology methods. The IBD variants were further screened in *in-house* whole exome data of 100 Saudi cohorts ensure their rare prevalence in the population.

**Results:**

Genetic screening has identified the digenic autosomal recessive mode of inheritance of *ITGAV* (G58V) and *FN1* (G313V) variants in IBD twins with early onset IBD. Findings from pathogenicity predictions, stability and molecular dynamics have confirmed the deleterious nature of both variants on structural features of the corresponding proteins. Functional biology data suggested that both genes show abundant expression in gastrointestinal tract and immune organs, involved in immune cell restriction, regulation of different immune related pathways. Data from knockout mouse models for *ITGAV* gene has revealed that the dysregulated expression of this gene impacts intestinal immune homeostasis. The defective *ITGAV* and *FN1* involved in integrin pathway, are likely to induce intestinal inflammation by disturbing immune homeostasis.

**Conclusions:**

Our findings provide novel insights into the molecular etiology of pediatric onset IBD and may likely pave way in developing genomic medicine.

## Introduction

Early onset (EO) IBD is a chronic autoimmune disorder of the gastrointestinal tract affecting children under the age of 6 years (1, 2). IBD is reported in almost all ethnic populations across the globe (1, 3). In particular, the global incidence of early onset IBD in pediatric age groups is increasing in the last 4 decades. This high incidence could be attributed to environmental changes and also to improvements made in the field of molecular diagnostics (4, 5). The EO-IBD patients from Middle Eastern population are often reported to have unique clinical characteristics compared to the patients from rest of the western world (6, 7). Crohn's disease (CD) and Ulcerative Colitis (UC) are the two most common forms of IBD, that distinct from each other based on the degree, and pattern of inflammation in the gastrointestinal tract. CD primarily affects the small and large intestines, besides inflammation of mouth, esophagus, stomach, and anus. It is characterized by skip lesions and transmural inflammation, whereas UC predominantly affects the colon and the rectum, and the lesions appear continuously through the rectum and the intestine. IBD patients may experience weight loss, vomitings, bowel obstruction, abscess, severe abdominal pain, and diarrhea (8). Treatment procedures usually focus on immunomodulators to maintain remission of symptoms. However, the patient may not always respond to the treatment, and face complications that require major abdominal surgery (1, 8).

Even though the exact IBD pathogenesis remains unclear, it is most likely caused by the dysregulation of the immune response triggered by unknown environmental factors in genetically susceptible individuals (4, 9). Manifestations of IBD in early life among children with EO-IBD underscores that, genetic defects are indeed the real disease causal factors. The monogenic etiology of EO-IBD due to molecular defects in *IL10RA/B,XIAP,CYBB, LRBA*, and *TTC7A* genes was initially reported (7). But the increased access to exome sequencing technology, which can effectively screen up to 200,000 exons, has rapidly expanded this list to 75 genes (10, 11). All of these new genes play a role in immune dysregulation (*IL-10, Foxp3, STAT1, and TRIM22*), T and B cell defects (*BTK, PIK3R1, ICOS, LRBA,IL21*, and *DKC1*), hyper and auto-inflammatory conditions (*CYBB,NCF,LACC1,SL37A4,ITGB2*), and epithelial barrier dysfunction (*ADAM17, COL7A1,GUCY2C,IKBKG,TTC7A*) (6, 10, 12).

The majority of the published reports on IBD are from non-Arab populations. However, the frequency of monogenic disorders in Arab populations is relatively high, in the Middle East, high consanguinity rates fuel the higher incidence of large number of monogenic disorders (13). Therefore, studying the rare familial cases of complex diseases in consanguineous Arab populations is expected to expand our knowledge about the molecular basis of disease. Over the years, numerous disease causal variants, genes, and pathways involved in the pathophysiology of complex genetic disorders are described (14, 15). Thus, the objective of the present study is to identify the causative genetic variants for EO-IBD in Arab patients. This study also aimed at elucidating the molecular relationship between the IBD causal genotype and the corresponding protein phenotype characteristics by using a variety of computational biology approaches.

## Materials and Methods

### Patient Recruitment, Clinical Evaluation, and Sample Collection

**Figure 1:**
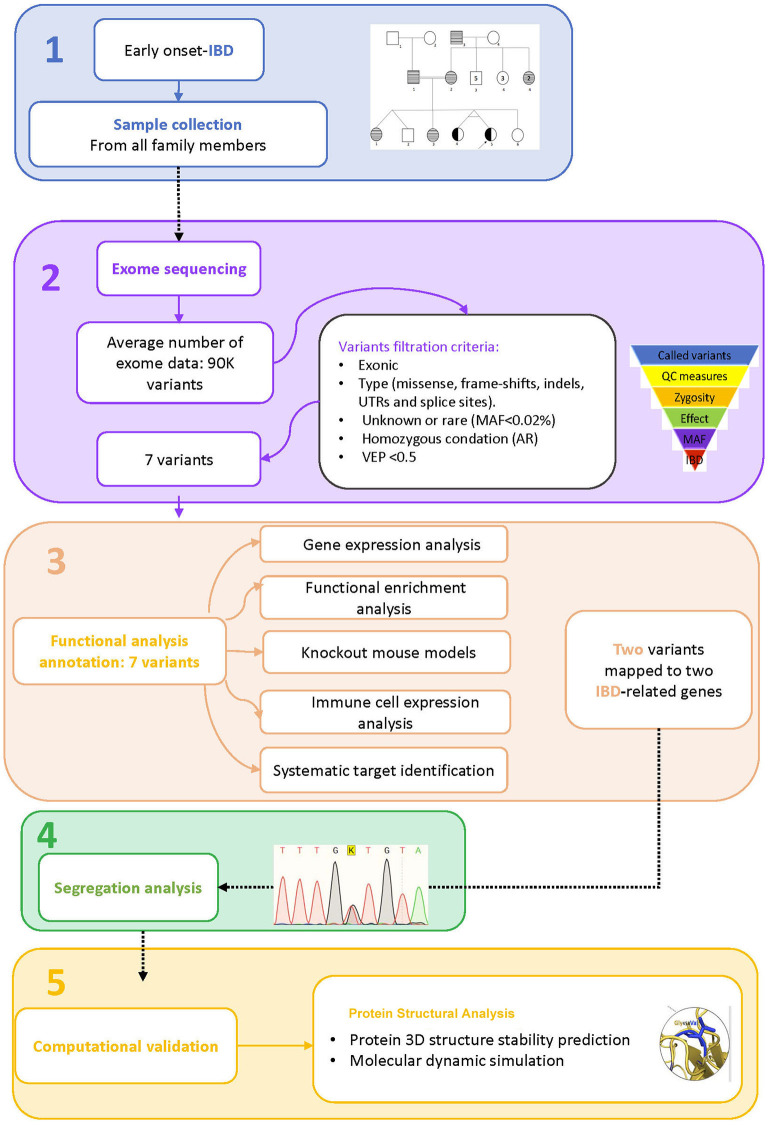
Study workflow. 1. Sample collection, 2. Exome sequence analysis, 3. Functional analysis annotation, 4. Segregation analysis, 5. Computational validation.

A consanguineous Arab family with monozygotic twins suffering from early onset inflammatory bowel disease (<2 years) was identified in the pediatric gastroenterology clinic at King Abdulaziz University Hospital. Both twins met the standard diagnostic guidelines of the European Society for Pediatric Gastroenterology, Hepatology, and Nutrition (ESPGHAN) for IBD (16, 17). The diagnostic workup consists of clinical examinations, intestinal endoscopy, and serological tests for anti-Saccharomyces cerevisiae antibodies (ASCA). A multi-generation pedigree was drawn with inputs from the parents. The Institutional Biomedical Ethics Committee for Human Research of King Abdelaziz University (KAUH), Jeddah, has approved this study according to standard international guidelines for medical research. EDTA tubes were used to collect 2.5 ml of blood from each member of the family for genetic analysis after they gave their consent to participate in this study. The general work-flow of the present study is presented in Figure 1.

### Genetic Analysis

#### DNA Extraction

The genomic DNA was extracted and purified with the QlAamp DNA blood kit (C# 51104) as per the standard manufacturer instructions. Nanodrop (ND-100 UV-VIS) spectrophotometer was used to measure the concentration and purity of the DNA. A 1% agarose gel electrophoresis was carried out to assess the integrity and purity of the DNA of all samples.

#### Whole Exome Sequencing and Candidate Gene Selection

The genomic DNA (100 ng/μl) of one proband, one healthy sibling, and both parents were used to prepare exome libraries using the Agilent Sure Select capture kit (Agilent Technologies, USA) as per CCDS and RefSeq databases. The HiSeq2000 Illumina platform was used to generate massive parallel short-read sequencing. All the sequencing reads were mapped against the human reference genome assembly (GRC38, NCBI) using BLAST (version 0.6.4d). GATK and SAM tools were used for base quality recalibration and variant calling such as SNPs and indels, respectively. A high-quality Phred score of >30 was set for reads on both strands to be included as potential variants. From WES data, variants, that passed the quality control metrics (Q30, read depth), mode of inheritance (autosomal recessive, *de novo*), pathogenicity effect (deleterious, non-deleterious), and minor allele frequency (MAF; >2%) were retained for further assessment (18). The allelic frequency of query variants was determined with the help of the Exome Aggregation Consortium (ExAC), 1,000 Genomes Pilot 1 dataset, Greater Middle East (GME) Variome Project, and the Saudi Human Genome Project (SHGP) (https://shgp.kacst.edu.sa/index.en.html). Then, we used the variant effect prediction tool (VEP) in Ensembl to determine the variant pathogenicity scores of the SIFT, Provean, and FATHMM tools.

#### Pathogenicity Characterization of Variants

##### Gene Expression and Functional Enrichment Analysis

The Ensembl (https://www.ensembl.org/index.html) database was used to determine the expression status of potential candidate genes across different tissues. From the output, we selected only the expression data of query genes (>0.5 TPM cut-off value) in the gastrointestinal tract, and immune organs. Furthermore, the Expression Atlas (https://www.ebi.ac.uk/gxa/home) was used to define the expression of potential candidate genes in IBD datasets. The differentially expressed genes were extracted based on logFC >1 and a *p*-value of <0.05. Moreover, the biochemical pathways of the selected genes were identified in the Ensembl browser and the Gene ID was provided as an input. The GO annotations were used to interpret the functional association of the 7 selected genes with autoimmunity and IBD.

##### Immune Cell Expression Analysis

The databases of Immune Cell Expression, Expression Quantitative Trait Loci (eQTL), and Epigenomics (DICE) provide the transcriptomic data generated from 15 immune cell types (subsets of T cells, B cells, monocytes, and NK cells). The gene ID was entered as an input to explore its expression status in different immune cells. The output was in the form of whisker box plots and pair-wise correlation graphs of individual genes showing > 1.5 fold expression changes (19).

##### Knockout Mouse Models

To understand the phenotypic and functional characteristics of the query genes, we used the Mouse Genome Informatics database (MGI) (http://www.informatics.jax.org/), which is a comprehensive resource of different mouse strains'- inbred and KO, biological data and analysis specifically designed. Input data was provided in the form of a gene symbol in the search box, and output refers to the physiological status of the mouse while the gene is knocked out (20).

##### Systematic Identification and Prioritization of Biological Targets for IBD

The Open Targets Platform (https://platform.opentargets.org/) is a webserver, which provides centralized access to a variety of computational tools for investigating the causation link, such as physical binary interactions, enzymatic reactions, or functional relationships between biomedical targets and illness/phenotypes. The query gene symbol was an input in the search box, and the output reflects the evidence score for each target-disease pair. The open target platform association score for the gene with a cutoff of >0.5 is deemed to predict to be a druggable molecular target (21).

##### Segregation Analysis and Exome Is the Validation

The segregation of potential disease causative variants in all the family members was determined by the dideoxynucleotide sequencing method. Briefly, we designed oligonucleotide primers using an open-source software PRIMER3; http://frodo.wi.mit.edu/. We made primer sets for two genes, one for query gene 1 and one for query gene 2. The forward primer for query gene 1 is 5′-ACTGGTGGTAGTTTGTTTACAG-3′and the reverse primer is 5′TAACTAGAAGAGAGGCTGGAA3′). The forward primer for query gene 2 is 5′TTGTCTTCTCTCACCCAGTT-3′ and the reverse primer is 5′-AGATAATGCATACCTGTCTC−3′). Both these primer sets were designed covering the variant locations on the corresponding chromosome. Then, gradient PCR was conducted to optimize the annealing temperature for primers (annealing temperature: 59°C). Following this, 35 cycles of PCR amplification were performed, and the success of the PCR was assessed using agarose gel electrophoresis before target purification and cycle sequencing on an ABI-Prism 3700 Genetic Analyzer (15). The rationale for the Sanger sequencing reaction was to confirm the segregation of causative mutations in this family and their mode of inheritance. All the sequence reads were analyzed by the BioEdit (https://bioedit.software.informer.com/7.2/) sequence alignment program. The variant position was determined by considering “A” of the first ATG codon of the mRNA. Moreover, the query variants were further screened in 100 in-house WES data that represents healthy Arab controls to validate their rarity.

##### Conservation Analysis

Conserved sequences refer to identical nucleotide or amino acid sequences among different organisms and species. In this study, the sequence conservation analysis option available in the Ensembl genome browser (https://asia.ensembl.org/index.html) was used to test whether the query variant falls into evolutionarily conserved regions in the orthologous genomes. The input was the query gene name and the output options included both the nucleotide alignment, showing the highly conserved regions in the protein, in addition, a phylogeny tree showing the relationship between different primates and humans at the query gene level was generated (22).

#### Protein Structure Analysis

##### Protein Modeling and Stability Analysis

The AlphaFold predicted structure was used to analyze the protein structure of the variant at molecular level. The AlphaFold, based on a machine learning approach, uses multi-sequence alignments to incorporate physical and biological knowledge about protein structure into the deep learning algorithm. The sequence of the candidate genes was collected from Uniport and used as the input for the modeling. As output, AlphFold generated the PDB coordinates based on local distance difference test model score (pLDDT). The pLDDT cutoff values for high quality >70 and <50 for poor quality structures. The AlphaFold also provides the “protected aligned error” graph, which elucidates each residue position error in the protein structure (23). The generated model is further visualized by molecular visualization tool PyMol (https://pymol.org/2/).

##### MD Simulation Analysis

The molecular dynamics characteristics of natural and variant protein versions were simulated using the CABS-flex 2.0 website (24). This website uses a coarse-grained modeling method paired with the reconstruction of anticipated structures to all atom representations to mimic the near-native dynamics of globular proteins. The CABS-flex methodology creates amino acid residue fluctuation profile. With 10 ns MD simulations, 100 cycles, and cycles between trajectory frames 50, the simulation time of CABS flex has been well adjusted to provide the greatest possible convergence. We provided the single -chain wildtype and mutant PDB coordinate files as an input. The output was obtained by an ensemble of protein models (all-atom resolution) reflecting the flexibility of both structures, in terms of 3-dimensional structural models (showing structure data), contact maps (showing residue-residue interaction pattern) and fluctuation plot (showing residue fluctuation profiles of the selected chain).

##### Structural Deviation and Stability Findings

YASARA molecular visualization tool was used to determine the structural deviation between energy optimized native and variant protein models. Two protein atomic coordinates were superimposed on each other, and then the corresponding RMSD values were estimated to quantify their structural similarity at both global and local residue levels. The cut-off RMSD values for variant-induced structure deviations at polypeptide chain and residue levels were >0.2 Å and >2 Å, respectively. The DUET web-based tool assesses stability of protein molecules upon the amino acid changes. The output contains different values of protein stability predictions in the form of Gibbs free energy (ΔΔG). The query variant destabilizes the protein when the value of the ΔΔG is negative. The PDB format or PDB ID of the native energy minimized protein structure, mutation information, and chain ID can be used as input.

##### Solvent Accessibility and Secondary Structure Analysis

The NetsurfP tool was used to predict the variant-induced changes in relative surface accessibility and secondary structural characteristics of query proteins. The amino acid sequence of query protein in FASTA format was the input for Netsurf. The output, this tool provides is the accessibility score (Z-score), which determines the buried or accessible nature of the amino acids (24). The NetsurfP also provides the graphical representation of secondary structural components (helices, sheets, and turns) of the protein.

## Results

### Clinical Presentation of IBD Patients in the Family

**Figure 2:**
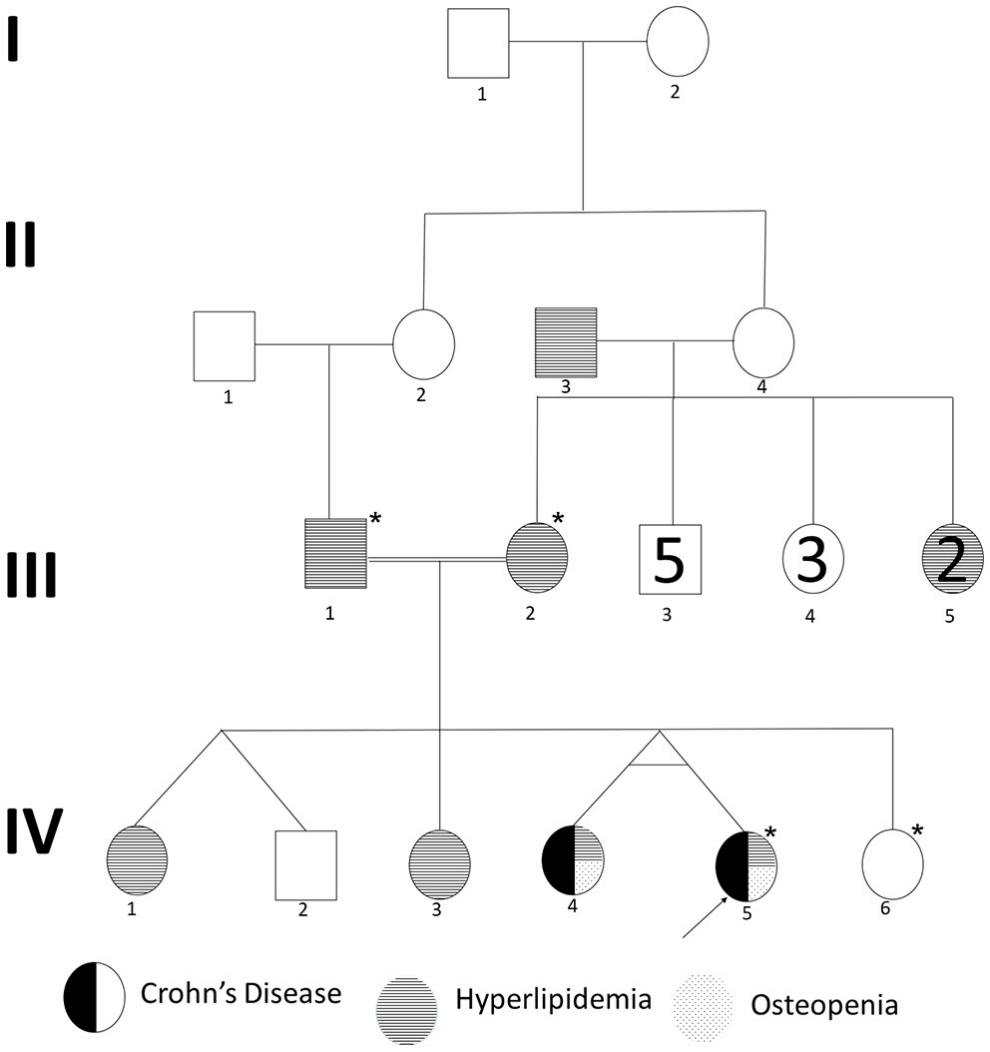
Pedigree of a consanguineous family with an autosomal recessive mode of inheritance of EO-IBD. Exome sequenced individuals are indicated with an star (*) mark. Proband is indicated by an arrow.

**Figure 3:**
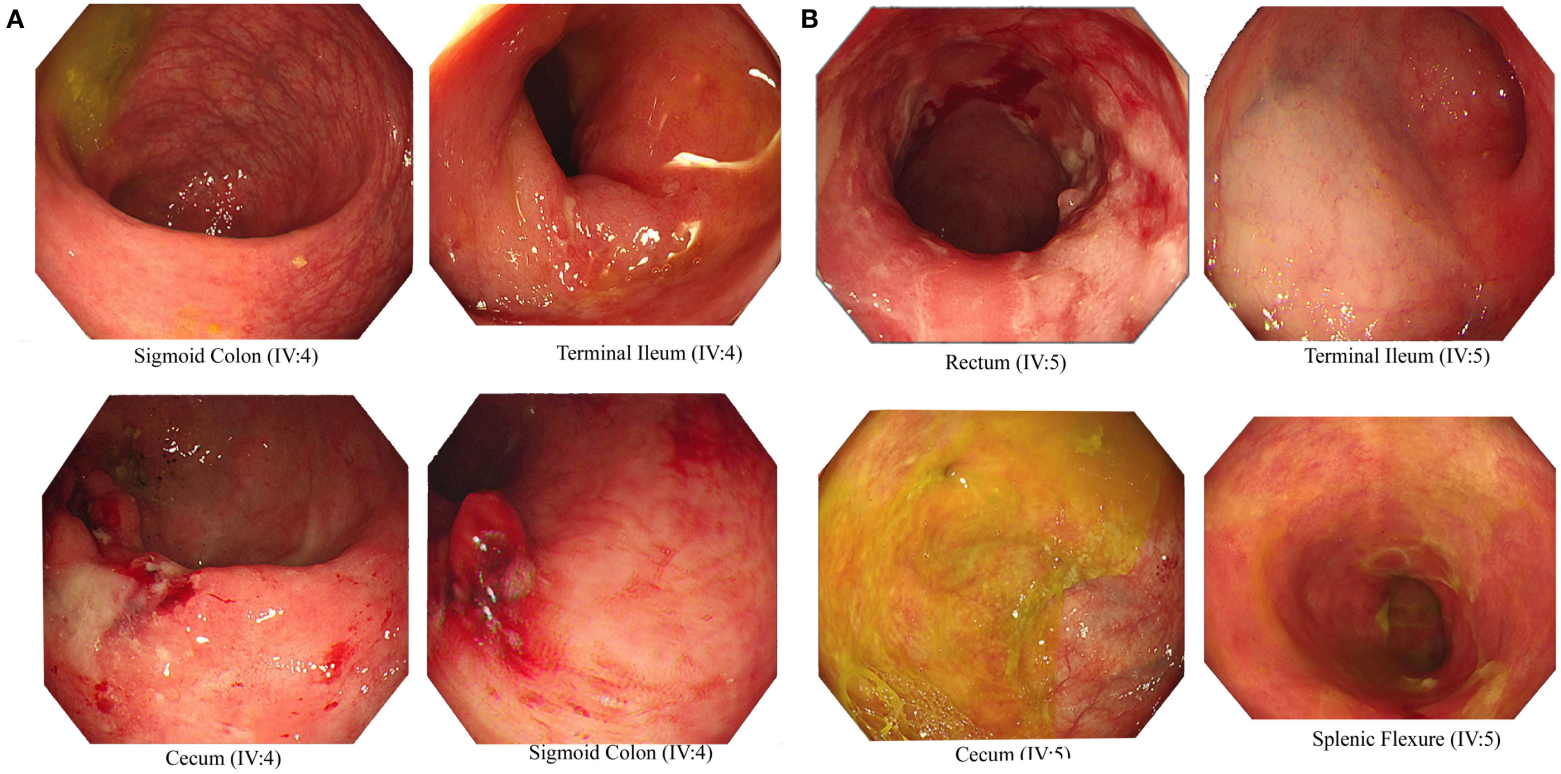
Endoscopic images representing Crohn's disease in the monozygotic twins. Inflammation and ulceration lesions **(A)** in sigmoid colon, terminal ileum, and cecum of IV-4 and **(B)** in rectum, terminal ileum, cecum, and splenic flexure of IV-5.

We recruited a consanguineous family with inherited familial EO-IBD (Figure 2). The probands are monozygotic twins born with an average normal weight of 2.5 kg. They initially presented with intermittent abdominal pain, fever, and bloody diarrhea at the age of 6 months. Laboratory stool results were positive for adenovirus. However, endoscopy and colonoscopy were not performed due to their age. At 18 months, gastrointestinal symptoms of the twins, including abdominal pain, bloating, and bloody diarrhea, became severe. They also exhibited mild osteopenia, eczema, severe skin rashes, weight loss, and lack of appetite. The endoscopy test showed fissures on the folds and the absence of intestinal villi. Serum tTG antibody testing has confirmed that both are CD positive. A series of colonoscopies over the years revealed that both twins displayed inflammation of variable severity (Figure 3). In one of the twins (IV4), the inflammation ranges from moderate to severe, characterized by altered vascularity, erythema, and friability. Shallow ulcerations were found as patches of various sizes surrounded by normal mucosa in the rectum, transverse and ascending colon, cecum, and terminal ileum. They were graded from moderate to severely damaged. In the second proband/twin, the localized inflammation is mild, as observed by the erosions and erythema & aphthous ulcerations in the terminal ileum. One of the twins (IV5) has undergone a series of surgical interventions due to the disease severity over the years. Histopathological changes and laboratory tests over the years suggest that both are at high risk of developing colon cancer in the future and need to be carefully monitored regularly by the multidisciplinary clinical team.

They are currently being treated with Pentasa (Mesalamine), which is a 5-Aminosalicylic Acid derivatives, and Imuran (Azathioprine AZA), an immunosuppressive agent, in addition to Remicade (Infliximab), a chimeric monoclonal antibody used to treat several autoimmune diseases, including IBD.

### Whole Exome Sequencing and Variant Filtrations

We performed whole-exome sequencing of four individuals in this family, including one of the proband (IV-4), a healthy sibling (IV-2), and both parents (III-1 & III-2) on the HiSeq2000/2500 platform with a 100-fold median coverage for 96% target regions. An average of ~95,593 variants were detected in each exome. Of these variants, 2,350 were novel ones. Non-coding variants (downstream, upstream, intergenic and intronic variants) except the regulatory region variants (promoter and 3'-UTR and 5'-UTR) were excluded. Filtering based on minor allele frequency (MAF <0.02) has resulted in approximately >1,697 variants per exome. Further filtering of the variants based on autosomal recessive (AR) mode of inheritance has shortlisted 18 potential variants. Computational pathogenicity predictions (SIFT score <0.01, Provean >0.5, and FATHMM score >0.5 predictions) have further reduced this number to 7 potential variants spanning in 7 genes (Table 1).

**Table 1:** List of potential candidate rare variants the in consanguineous family with EO-IBD.

**Genes**	**Chr**	**Position**	**rs ID**	**CDS position**	**Amino acid position**	**Type of mutation**	**MAF**		**Variant Effect Predictor**		
							**genomAD^**a**^**	**GME^**b**^**	**PROVEAN^**c**^**	**SIFT^**d**^**	**FATHMM^**e**^**
ITGAV	2	186,649,861	N/A	c.1373G > T	p.Gly458Val	Missense	NA	NA	0.9595	0	0.99384
FN1	2	215,425,192	rs143118391	c.938G > T	p.Gly313Val	Missense	0.000004	NA	0.79571	0	0.94163
CORIN	4	47,807,021	rs370769751	c.90G>A	p.Met30Ile	Missense	0.000004	NA	0.3735	0.01	0.82393
ACSM2A	16	20,480871	rs751630877	c.1459G > T	p.Ala487Ser	Missense	0.000016	NA	0.45404	0.05	0.87066
LDLR	19	11,113,346	rs879254847	c.1255T > G	p.Tyr419Asp	Missense	NA	NA	0.9876	0	0.94345
LRRC8E	19	7,899,291	rs764883772	c.769C > T	p.Arg257*	stop_gained	0.000016	NA	NA	NA	0.88096
HLA-DQB1	6	32,666522	rs35560667	c.85_86insGG	p.Leu29fs	Frameshift	NA	NA	NA	NA	NA

a *genomAD(The Genome Aggregation Database) =;*

b *GME(Great Middle East) =;*

c *PROVEAN= <0.5 = Benign, >0.5 = Deleterious;*

d *SIFT >0.05 = Benign, <0.05 = Deleterious;*

e *Fathmm= <0.5 = Benign, >0.5 = Deleterious; NA, Not available*.

### Functional Annotation of Screened Variants

#### Transcriptomics Data Analysis

**Figure 4:**
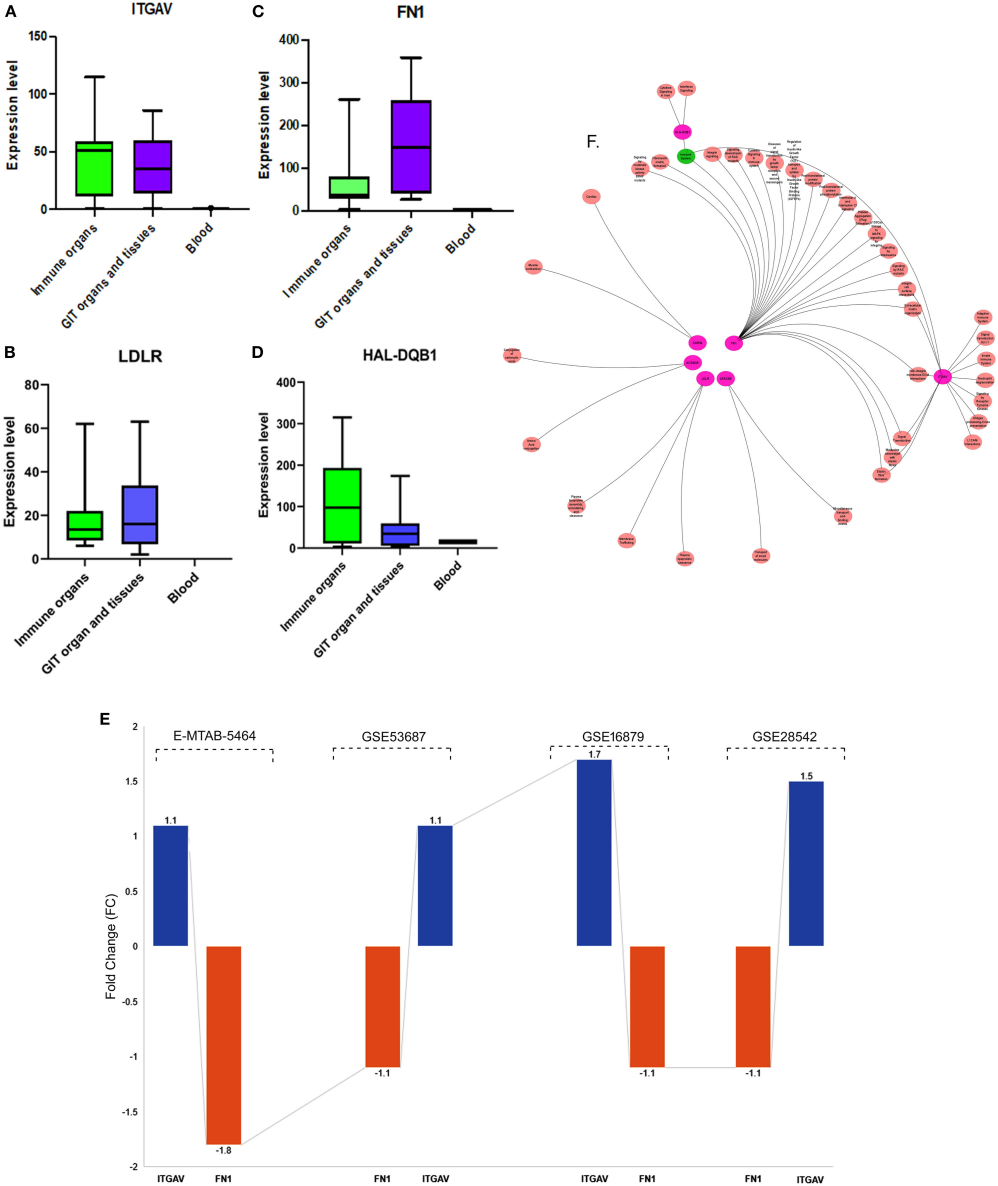
**(A–D)** Ensembl based transcriptomics of potential causal genes across different tissues and organs. Gastrointestinal tract includes small intestine and duodenum tissue, and small intestine Peyer's patch. Immune organs and cells include leukocyte, spleen, lymph node, thymus, EBV-transformed lymphocyte, and bone marrow, and blood. Transcription levels are in FPKM (fragments per kilobase of exon model per million mapped reads) and TPM (transcripts per million). Transcription scale: low (0–10), medium (11–1000), and high (>1000). **(E)** Ensembl functional annotations of *ITGAV* and *FN1*gene*s*. **(F)** Pathway enrichment of the 7 candidate genes (in pink) only *HLA-DQB1, ITGAV* and *FN1* are involved in immune related pathways.

The 7 highly pathogenic variants selected from exome data were further screened by diverse computational biology approaches, like gene expression, genotype-phenotype correlations in knock-out mouse models, and immune cell expression analysis. In this context, the Ensembl based transcriptomics data analysis of the 7 genes (harboring 7 variants) has shown the significant expression (TPM of > 40) of 4 (LDLR, HAL-DQB1, ITGAV, and FN1) in gastrointestinal organs (small intestine, duodenum, and small intestinal Peyer's patches, colon, and large intestine) and immune organs (leukocyte, spleen, lymph node, thymus, EBV-transformed lymphocyte, and bone marrow) (Figure 4).

#### Pathway Enrichment Analysis

The Ensembl analysis of the 7 query genes has confirmed the significant enrichment of four genes (*CORIN, ACSM2A, LDLR*, and *LRRC8E*) in cardiac-related pathways like cardiac conduction and Muscle contraction (R-HAS-5576891), miscellaneous transport and binding event (R-HAS-5223345), plasma lipoprotein clearance (R-HSA-174824), LDL clearance (R-HAS-8964034), and Catherin-mediated endocytosis (R-HAS-8856828). The remaining three genes, *HLA-DQB1, ITGAV*, and *FN1* are involved in immune-related pathways (Figure 4). The *ITGAV* significantly plays an essential role in the innate immune system (R-HAS-168249), adaptive immune system (R-HAS-1280218), antigen processing presentation (R-HAS-1236975), Class I MHC mediated antigen processing (R-HAS-98316). The *FN1* gene link to the interleukins pathway (RHSA-6785807), cytokine signaling in the immune system (R-HAS-1280215), integrin signaling (R-HSA354192), and fibronectin matrix formation (R-HAS-1566977).

#### Immune Cell Specific Enrichment of IBD Genes

**Figure 5:**
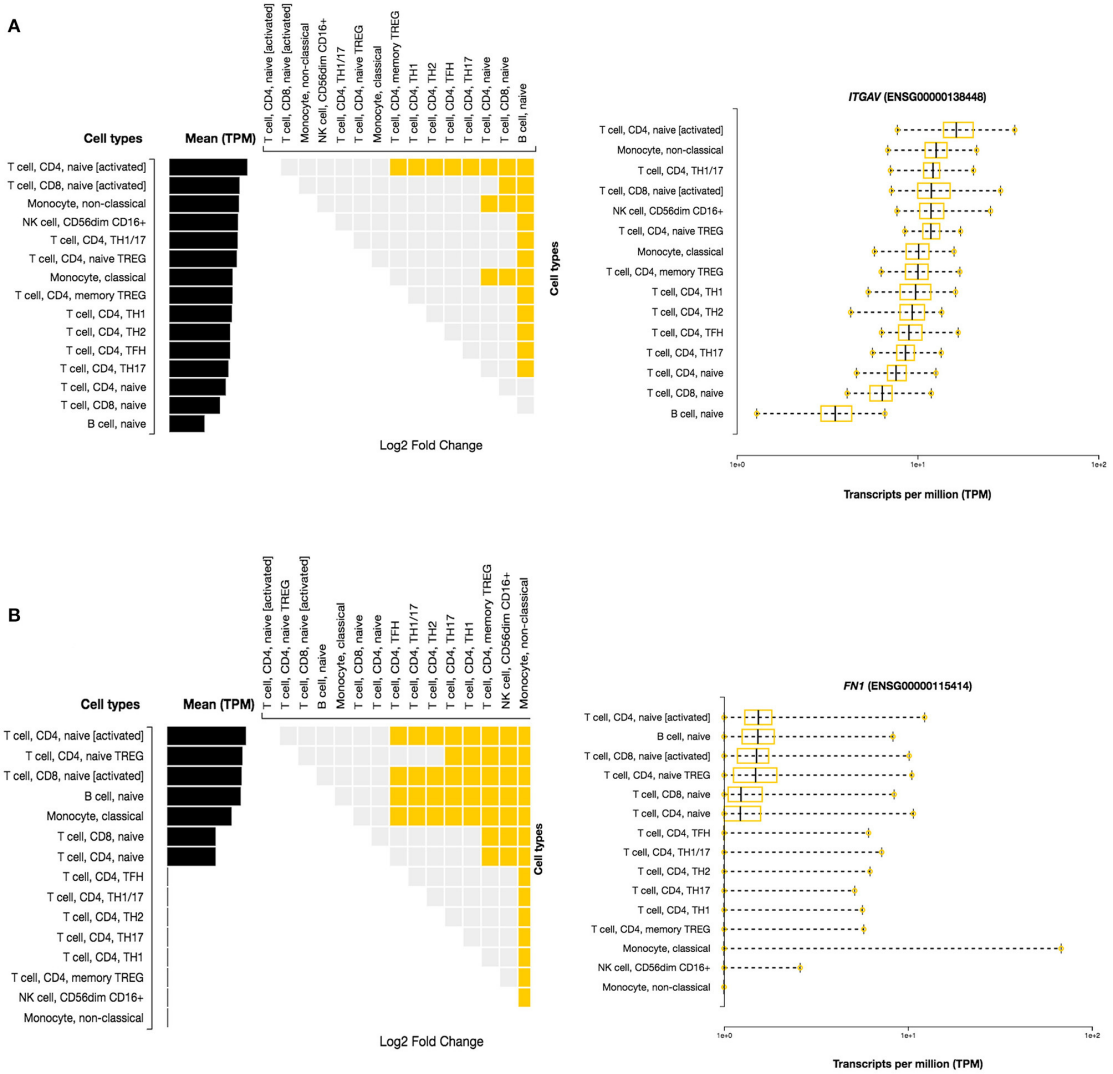
Differential Expression of *ITGAV* and *FN1* in DICE Database of Immune Cell-specific Gene Expression. **(A)** *ITGAV* gene is enriched significantly in naïve B-cell, CD4^+^ T cell types (TH17,TFH1,TFH2) immune cell types. **(B)** *FN1* enrichment is seen in monocytes and CD4^+^ T cell types.

Here, we investigated the immune cell type enrichment of the seven candidate genes, of which only LDLR, HLA-DQB1, ITGAV, and FN1 have shown significant findings (Figure 5). The HLA-DQB1 gene is enriched in CD4+ T cell types, including the following immune cell types: TH2, TH17, TREG, monocytes, and naïve B-cell. The ITGAV and *LDLR* genes are enriched significantly in naïve B-cell, CD4+ T cell types (TH17, TFH1, TFH2), immune cell types (logFC 2.34). The highest enrichment of FN1 was seen in monocytes and CD4+ T cell types with logFC 4.29 (*p* < 0.001).

#### Knockout Mouse Models

**Figure 6:**
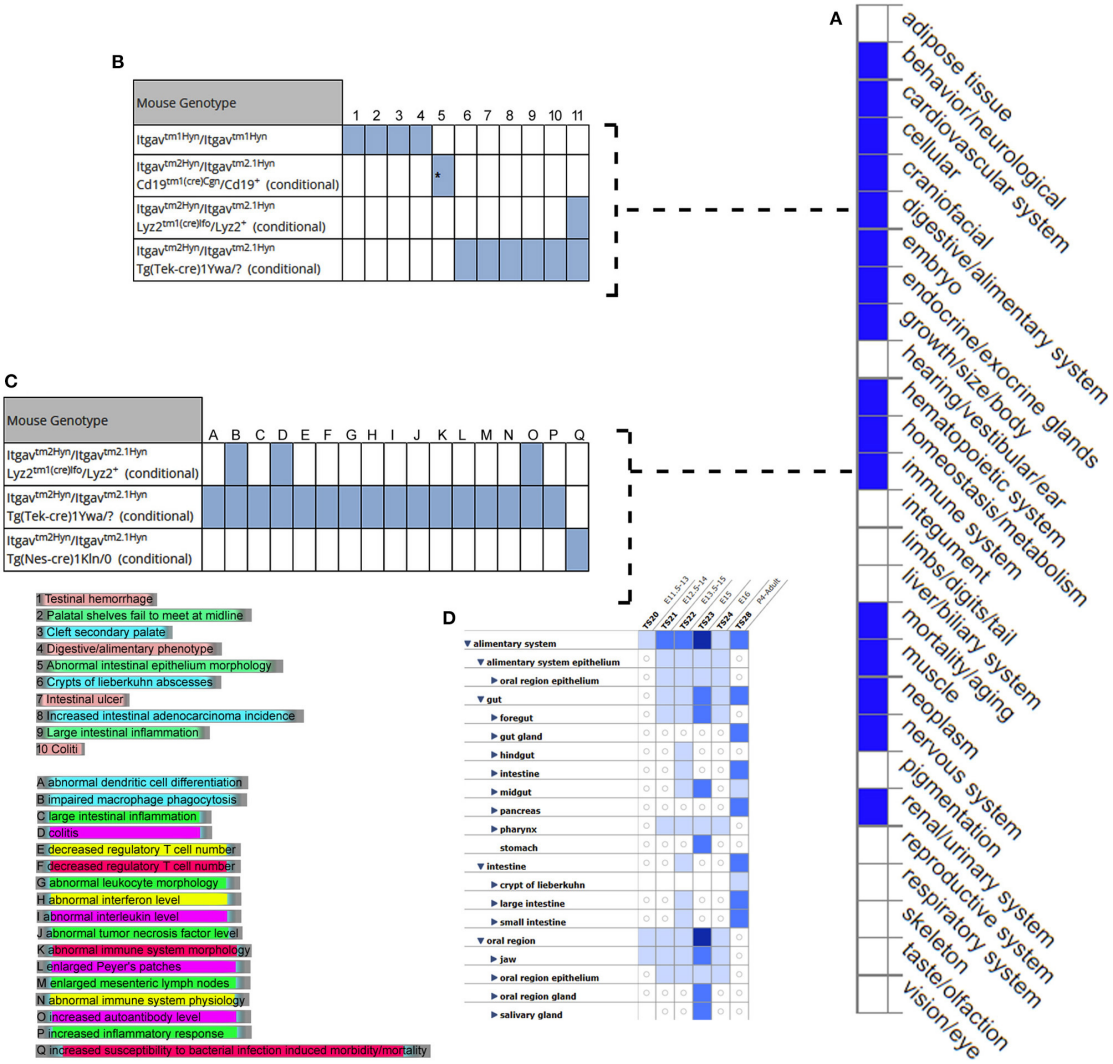
Expression and phenotype characteristics demonstrated by *ITGAV* knockout mice from Mouse Genome Informatics (MGI) database. **(A)** System level Mammalian phenotype effect are displayed. Blue boxes represent abnormal phenotypes and blank boxes represent the normal phenotype for *ITGAV* mutants in that system. **(B,C)** Tables represent the most genotype affect relevant pathways in **(B)** digestive/alimentary system **(C)** immune system. **(D)** *ITGAV* expression in alimentary canal system. Shades of blue color in the grid indicate the detection of the variable expression level, dark blue > 50, blue 5–10, light blue 1–4 annotations.

MGI database had the phenotype data available for only 3/7 (42.8%) query genes, including *LRRC8E, LDLR*, and *ITGAV* for knockout mouse models (Figure 6). The knockout mouse model of *LRRC8E* has demonstrated the immune system phenotype like abnormal circulating cytokine level, decreased circulating interferon-beta level, increased susceptibility to viral infection and decreased interferon-beta level (MGI:1919517) (Lrrc8e^em1Bcgen^). *LDLR* knockout mouse models exhibited immune system phenotype like increased macrophage derived foam cell number, decreased circulating HDL cholesterol level, increased circulating cholesterol levels (MGI:3622102) (Ldlr < ^tm1Her>/^Ldlr ^tm1Her^). *ITGAV* was detected in the gastrointestinal tract with score between (5 and 50) number of annotations. The major phenotype characteristics observed for *ITGAV* mouse models were intestinal hemorrhage, colitis, large intestinal inflammation, intestinal ulcer, abnormal interferon level, abnormal interleukin level, and abnormal immune system (MGI:96608) (itgav^tm2Hyn^).

#### Open Target Platform

In the Open Target Validation Platform analysis, the seven genes demonstrated the genotype-phenotype association score of >0.002. Of those 7, only ITGAV, FN1, HLA-DQB1 showed the disease phenotype related to the immune system and gastrointestinal tract with a score of >0.26. Tractability information for ITGAV, and FN1, reveals the availability of small molecules, antibodies, PROTAC, and other modalities. The ITGAV is predicted to be targeted by antagonists, inhibitors, or binders classes, consisting of drugs (ABITUZUMAB, STX-100) and monoclonal antibodies (INTETMUMAB, ETARACIZUMAB, TN-161), which are currently undergoing phase I clinical trials. The second gene, FN1, is targetable by a binder (L19IL2, L19TNSA, AS-1409) and proteolytic enzyme class drugs (OCRIPLASMIN), which are currently undergoing phase I clinical trials (Table 2).

**Table 2:** Open target platform for drug analysis.

**Gene**	**Geno-pheno association**	**Known drugs**	**Action**	**Phase**	**Tractability predictions**			
					**Small molecule clinical precedence**	**Antibody clinical precedence**	**PROTAC**	**Other modalities (Enzyme, peptide, oligonucleotide etc.,)**
*ITGAV*	26	CILENGITIDE	antagonist	>1	+	+	+	+
		STX-100	Inhibitor	>1				
		ABITUZUMAB	Inhibitor	>1				
		INTETMUMAB	antagonist	>1				
		ATN-161	antagonist	>1				
		ETARACIZUMAB	antagonist	>1				
		IMGN-388	Binding	1				
*FN1*	53	OCRIPLASMIN	Proteolytic enzyme	>1	+	+	+	+
		L19IL2	Binding	>1				
		L19TNSA	Binding	>1				
		AS-1409	Binding	1				
		L19SIP131I	Other	>1				
*HLA-DQB1*	38				-	-	-	-

*Note: +, Data Available; −, Data Not Available*.

#### Prioritization of IBD Candidate Genes

Based on the diverse systems biology and functional annotations of the 7 query genes, ITGAV, FN1, and HLA-DQB1 were prominent in autoimmune diseases. HLA-DQB1 was eliminated from further downstream analysis because it is a known prerequisite genetic factor involved in developing autoimmune diseases like celiac disease and rheumatoid arthritis. Thus, the remaining two genes (ITGAV and FN1) were prioritized for further analysis. The EMBL-EBI Expression Atlas data on these two genes confirmed the overexpression of ITGAV (logFC1.1 with *P*-value <0.04) and downregulation of FN1 (logFC-1.1 with *P*-value <4x. 10-6) genes in IBD patients (Figure 4), providing the evidence for their causal role in IBD pathogenesis (Figure 4). Furthermore, the *in-house* WES data confirmed the absence of *ITGAV* and *FN1* variants in 100 Arab healthy controls.

### Sanger Sequencing and Conservation Analysis

The Sanger sequencing of ITGAV (c.1373 G > T) FN1 (c.938 G > T) findings have confirmed that both monozygotic twins (III.3&III.4) were homozygous for minor allele. In contrast, parents were heterozygous carriers (III.1&III.2), siblings were either heterozygous (III.1, III.3, III.6) or homozygous (III.2) for the normal allele. All the exonic, splicing, missense, and frameshift variants showing compound heterozygous inheritance mode had a high population frequency (MAF > 0.02) and were neutral in their impact. The segregation analysis confirmed the absence of these variants in 100 Arab healthy control cases.

**Figure 7:**
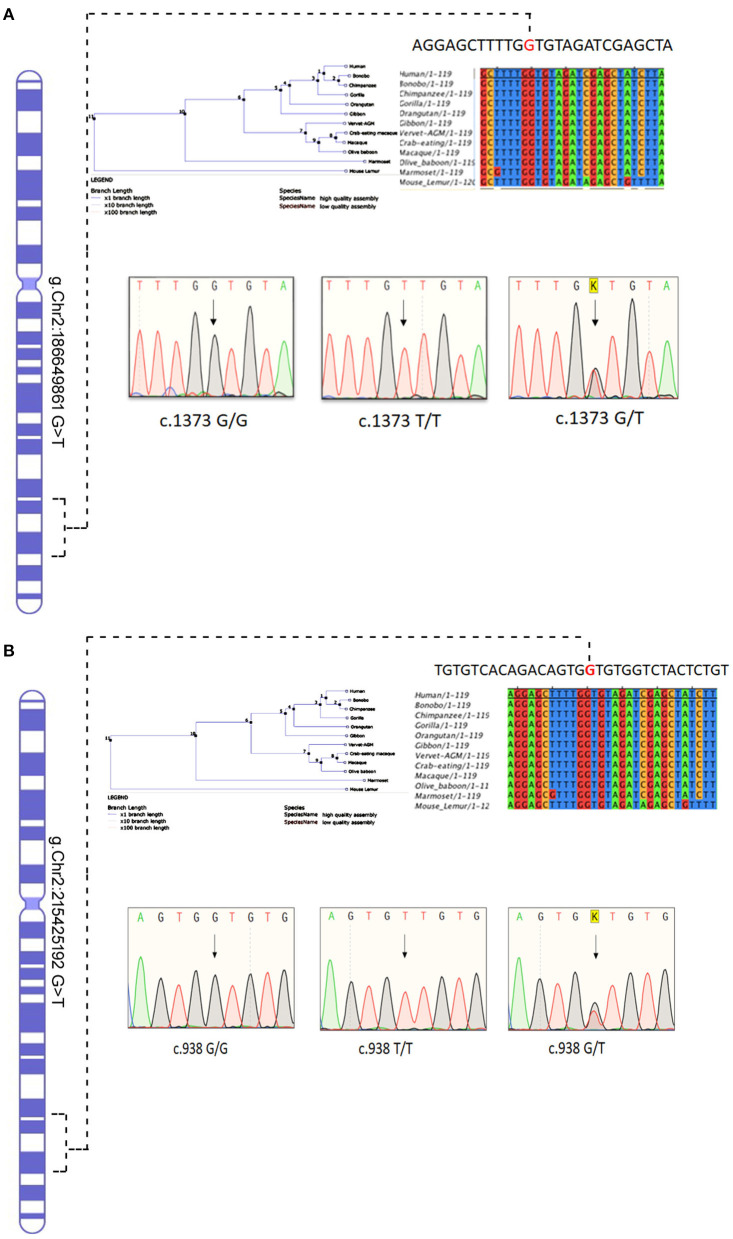
Chromosomal location of human **(A)** *ITGAV and* **(B)** *FN1 genes* in chromosome 2 and chromatograms of both missense mutation sequence (wild type, heterozygote, and homozygous mutant genotypes) observed in the family. The twins are homozygous for both mutations *ITGAV* (c.1373G/G), *FN1*(c.938 G/G). Parents are heterozygous for both mutations. Nucleotide sequence Alignment and phylogenetic tree of human *ITGAV* and *FN1* genes.

The conservation analysis has predicted that both ITGAV (c.1373 G > T) FN1 (c.938 G > T) variants are in the highly conserved gene regions in both humans and 12 other primates, i.e., Gorilla, Chimpanzee, Bonobo, Homosapien, Orangutan, Gibbon, Mouse Lemu, Marmoset, Vervet AGM, Olive Baboon, Macaque and Macaque CE, and are strongly predicted to have a negative impact on the functions of corresponding proteins (Figure 7).

### Downstream Proteins Analysis

#### 3D Structural Characterization of ITGAV and FN1 Missense Variants

**Figure 8:**
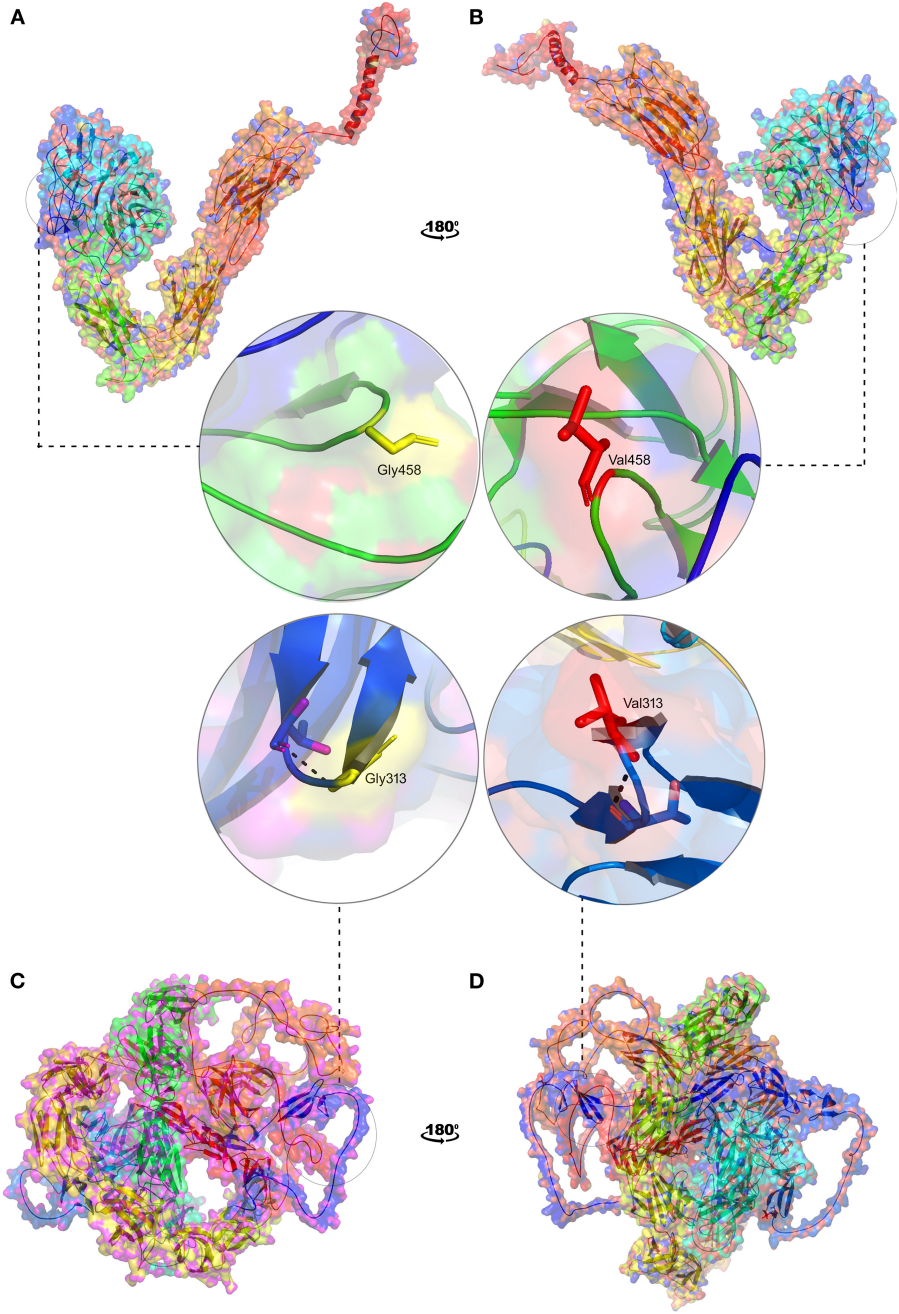
3D structural representation of *TGAV and FN1* wild and mutant types. Magnified structures of **(A)** *ITGAV* (Gly458) wild type in yellow **(B)** *ITGAV* (Val458) mutant in red and **(C)** *FN1* (Gly313) wild type in yellow **(D)** *FN1* mutant (Val 313) in red.

In the absence of the availability of 3D structure of ITGAV and FN1, we initially built high-resolution protein structures with the help of AlphaFold. The company used the AI and neural network to develop the structures for almost all genes), by applying the state-of-the-art neural network-based methodology. It is the first computational approach demonstrating accuracy comparable to the experimental designs in most cases and significantly outperforming other methods. The total length of human ITGAV protein chain A structures (1048aa), with model confidence (pLDD > 90) downloaded as a PDB file. The full length of human FN1 protein chain A structure model (2477aa), with model confidence (90 > pLDD > 70), was downloaded as a PDB file. The full-length tertiary models were subsequently processed for energy minimization and stereochemical assessment steps as described [15]. Then, using this energy minimized native protein structures, the mutant versions of ITGAV (G458V) and FN1(G313V) were modeled using homology modeling by the SWISS-MODEL approach (25) (Figure 8).

#### Protein Stability Analysis

The pathogenic amino acid substitutions can cause changes in free energy values, thus influencing protein stability directly. Herein, we analyzed the impact of ITGAV (G458V) and FN1 (G313V) on protein stability by DUET methods (Table 3). The DUET method incorporates graph-based signatures to estimate the impact of mutations based on atomic distance patterns surrounding amino acid residues in the protein. It also predicts the deleteriousness of amino acid substitutions by estimating the free energy differences between the native and mutant protein structures in both folded and unfolded states. The superpose analysis of wild and mutant ITGAV, and FN1 protein structures revealed no significant difference (<0.078 Å; 0.059 Å) on the whole structure. However, a significant deviation (>0.345 Å; >0.2 Å) was observed at residue RMSD level of wildtype and mutant models of *ITGAV* and *FN1*, respectively. The relative surface accessibility and secondary structural components of the amino acid residue of ITGAV were exposed in the wild type and buried in the mutant type however, in *FN1* no significant change in the surface accessibility and secondary structural. According to the DUET method, both ITGAV (-0.0112kcal/mol) and FN1(-0.204Kcal/mol) variants were destabilizing to the corresponding proteins owing to their negative free energy values (ΔΔG).

**Table 3:** DUET program protein stability prediction of *ITGAV* and *FN1* variants.

**Gene**	**Variant**	**DUET** **predicted stability change (ΔΔG)**	**Solvent accessibility**	**Structural deviation** **(RMSD)**	
				**Whole structure level (Å)**	**Residue level (Å)**
*TGAV*	G458V	−0.112 kcal/mol (Destabilizing)	E > B	0.078	0.345
*FN1*	G313V	−0.204kcal/mol (Destabilizing)	B > B And the secondary structures change	0.059	0.2

*E, Exposed; B, Buried; DUET Positive score, Stabilizing; Negative score, Destabilizing*.

#### Molecular Dynamics Simulations

From the MD simulation output, an interactive a line graph shows C-alpha root means square fluctuation (RMSF) values of the native and mutant amino acid residues of *ITGAV* and FN1 protein. In the solvent system, the fluctuation values (Å) were calculated based on the rigid and flexible nature of amino acid residues of the *ITGAV* and *FN1* protein (Supplementary Figure 1). In the ITGAV wildtype model, helix 6 (995-1017 aa) and coil 4, coil 21, coil50, coil56, coil 63 are showing RMSF of 4-8 Å. The 64 beta sheets, 58 coils and 5 helices are showing less RMSF. However, we found no significant changes in the secondary structural components of the mutant proteins during simulations. Though, the mutation G458V of *ITGAV* is localized in 28^th^ coil and altered its flexibility from 0.79 to 0.484 from Å (Supplementary Figure 2).

The *FN*1 wildtype model (1-500 residues) 3 helices and coil 26, coil 30, coil 48, coil 49 and 46 beta sheet are showing amino acids as rigid and the 3,5 beta sheets showing flexible amino acid between 2 and 4 Å. The mutation G313 of *FN*1 is localized in 29^th^ coil and altered its flexibility from 1.19 to 1.895 from Å (Supplementary Figure 3).

## Discussion

EO-IBD patients present a distinct disease progression and pathogenesis differing from adult IBD, in terms of variability of clinical presentation, resistance to conventional immunosuppressant therapy, and unique complications (26, 27). Unlike other forms of IBD, EO-IBD is an aggressive disease with strong family history of the disease and has a causal monogenic factor often involving genes associated with primary immunodeficiency, owing to inherited variants that may contribute to dysregulated immunologic homeostasis in the intestine (1). Soon after the report on the involvement of defective *IL10* signaling system in EO-IBD, numerous studies conducted on Caucasians, Asians, Africans have quickly expanded the total gene list to more than 50 genes, (28). Incidence of pediatric or EO-IBD cases in Saudi Arabia was found to be 0.59 per 100,000, reflecting underreporting of many cases (29). Therefore, molecular diagnosis of all suspected EO-IBD with high throughput technology like whole exome sequencing is essential for early identification, optimization of treatment outcome, family screening and genetic counseling of the patients and their family members.

In this report, we used WES to identify digenic causal variants in consanguineous IBD family with monozygotic twins following the autosomal recessive mode of inheritance. By systemic application of comprehensive systems biology approaches, we narrowed down the potential gene list for EO-IBD to allelic variants in ITGAV (G458V) and FN1 (G313V) genes, which are involved in a range of immune cell activities, including T cell and regulatory pathways, as well as B cell development pathways. The first gene, ITGAV is located on the long arm of chromosome 2 (2q32.1), encoding a 1048 aa long integrin alpha protein chain, which forms a transmembrane receptor protein, connecting the cytoskeleton with the extracellular matrix (EMC), including collagens, fibronectins, and other cellular receptors. A GWAS study identified 21 new loci, three of them were located within 150 kb of integrin gene (ITGA4, ITGAV, and ITGB8) cluster, and one overlapped with a fourth integrin gene, *ITGAL*. Integrins are bidirectional signaling cell adhesion mediators that play an essential role in leukocyte homing and cell differentiation in inflammation and cancer. ITGAV is a potent activator of transforming growth factor (TGF) with a range of cell-type-specific effects. The G458V variant is located 19 AA upstream of integrin alpha domain, which spans between 467 and 914 th AA in ITGAV protein. The proximity of pathogenic variants to functional domain may impact the folding, dynamics behavior, and stability of the candidate proteins. The amino acid residue level structural deviation identified in ITGAV (G458V) is likely to disturb the structural features in the protein. ITGAV predicted stability change were destabilizing (-0.0112kcal/mol). This stability changes in ITGAV protein mainly impact the main biological functions such as calmodulin-binding, actin-dependent ATPase activity, calcium-dependent protein binding, and microfilament motor activities (30). In mice studies, alpha integrin plays a significant role in maintaining intestinal immune homeostasis by increasing TGF-b and Treg differentiation (31). Deleting alpha integrin in the myeloid cell or dendritic cells resulted in spontaneous colitis via failure of a generation of Treg cells and apoptotic cells and the reduction of TGF-B in the intestine. Therefore, integrin may maintain the homeostasis of the intestinal immune system by mediating both TGFB and IL-10 signaling pathways (31).

The second gene, Fibronectin1 (*FN1)* is located on the long arm of chromosome 2 (2q35) and encodes a high molecular weight (~500-~600 kDa) protein with 2477 aa residues. It is a fibronectin glycoprotein of the extracellular matrix, binds to membrane receptors called integrins and also binds to extracellular matrix protein like collagen and fibrin. It played an important role in cell migration, adhesion, proliferation, hemostasis, and tissue repair (32). One deleterious mutation reported in the *FN1* may be related to the IBD- associated colorectal cancers (33). The G313V variant is located on fibronectin type I domain, and it spans from 308^th^ to 345^th^ AA in *FN1* protein. The amino acid level structural deviation identified in *FN1*(G313V) is likely to disturb the structural features in the protein with a negative free energy value of (0.204Kcal/mol). This stability changes in *FN1* protein possibly impact its main biological functions such as calmodulin binding, actin-dependent ATPase activity, calcium-dependent protein binding, and microfilament motor activities.

Digenic mechanisms for many genetic diseases are reported; a study on familial hematuria found digenic inheritance in one family presenting with FMH, renal failure/ESRD, FSGS and cystic kidneys, can clearly explain the phenotypic spectrum than one gene alone. Even though the digenic mechanisms have not been identified in the field of IBD, implementing this digenetic inheritance of *ITGAV* and *FN1* mutations mapped to the same chromosome in this family can explain the severe spectrum of the symptoms in those patients, but further investigation is needed to confirm their potential causal role (34, 35).

Interestingly, both *ITGAV* and *FN1* are commonly involved in integrin pathway which plays pivotal role in maintaining intestinal immune homeostasis by increasing TGF-b. The role of TGF-b which is activated in the human intestine to control immunity is completely unknown. Recent evidence suggests that targeting the TGFb pathway in IBD may be beneficial to some patients. Here, we find that the TGFb-activating integrin, *avb8* is expressed on human intestinal CD1c DC, and that expression is increased on this DC subset in patients with CD. Integrin *avb8* expression was also increased on DC after *ex vivo* treatment with LPS, which enhanced their ability to activate TGFb, and *avb8* expression promoted induction of *FOXP3* expression in naive human T cells (1, 31).

Taken together, our study uncovers a new pathway in which the TGFb-activating integrin *avb8* is expressed on human intestinal DC and it is upregulated in patients with CD. This study has some limitations. Due to the small sample size, we focused only on the known primary immunodeficiency pathways, which are excellent candidates for EO-IBD. However, IBD is a complex disease, mutations in this cohort influence genes and pathways that are similarly essential but have not yet been investigated; despite these limitations, this mode of analysis allows us to understand the complex genomics of EO-IBD. It may aid in the identification of disease pathways in this population. It is now widely recognized that an immunologic evaluation is crucial in children under the age of five with IBD. A systematic strategy to evaluate the disease pathogenesis has been suggested to understand the etiology of disease in this population. As a result, a framework for detecting potential immunological abnormalities is key to identifying the novel therapy and planning for better clinical management of EO-IBD patients worldwide. We propose that patients with EO-IBD undergo a more comprehensive immunologic examination.

In summary, significant findings of functional rare variants in novel genes in *ITGAV* and *FN1* provide strong support for the roles of integrin and fibronectin in early onset IBD. Hence, many of our findings will provide insight into disease mechanisms and targets for biological experiments to further understand their role in IBD pathogenesis. However, other deep sequencing approaches (e.g., whole-genome, target gene resequencing) will be needed to identify variants in non-coding regions and the contribution of structural variants (e.g., larger insertions and deletions, copy number variants, etc.) to IBD risk.

## Data Availability Statement

The datasets for this article are not publicly available due to concerns regarding participant/patient anonymity. Requests to access the datasets should be directed to the corresponding author.

## Ethics Statement

The study was conducted according to the guidelines of Institutional Review Board (IRB) protocols at King Abdulaziz University Hospital. Written informed consent to participate in this study was provided by the participants' legal guardian/next of kin.

## Author Contributions

BB, RE, and HA-N: conceptualization and methodology. HA-N and BB: software, visualization, and formal analysis. HA-N, OS, BB, RE, AM, and NS: investigation. BB and NS: resources. HA-N, NS, and RE: writing—original draft preparation. BB, RE, NS, OS, AM, and HA: writing review and editing. NS, NA-S, BB, and RE: supervision. BB: project administration and funding acquisition. All authors contributed to the article and approved the submitted version.

## Conflict of Interest

The authors declare that the research was conducted in the absence of any commercial or financial relationships that could be construed as a potential conflict of interest.

## Publisher's Note

All claims expressed in this article are solely those of the authors and do not necessarily represent those of their affiliated organizations, or those of the publisher, the editors and the reviewers. Any product that may be evaluated in this article, or claim that may be made by its manufacturer, is not guaranteed or endorsed by the publisher.
