# Supplementary material for: Exome Sequencing Identifies the Extremely Rare ITGAV and FN1 Variants in Early Onset Inflammatory Bowel Disease Patients
Source: Front Pediatr. 2022 May 26;10:895074. doi: 10.3389/fped.2022.895074 (PMC9178107; doi:10.3389/fped.2022.895074)
Supplement: Supplementary file 1 [file Data_Sheet_1.docx]

**Supplementary Figure S1**

**Figure S1: : CABS-flex MD simulation of *ITGAV* & *FN1* wild and mutant type models, the ribbon view represents the Wildtype and mutant simulation model.** A&B. represents the *ITGAV* wildtype and mutant simulation model respectively. C&D. represents the *FN1* wildtype and mutant simulation model respectively.

**Supplementary Figure S2**

**Figure S2: Molecular dynamics simulation of *ITGAV* mutant and wild type,** **an interactive 2D plot presenting residue-wise fluctuations recorded throughout the simulation.** The RMSD value of *ITGAV* wildtype models demonstrated high degree of flexibility when compared to mutant protein.

**Supplementary Figure S3**

**Figure S3: Molecular dynamics simulation of *FN1* mutant and wild type, an interactive 2D plot presenting residue-wise fluctuations recorded throughout the simulation.** The RMSD value of *FN1* wildtype models shows minimum fluctuation compared to mutant protein.
